# The gender suicide paradox under gender role reversal during industrialisation

**DOI:** 10.1371/journal.pone.0202487

**Published:** 2018-08-23

**Authors:** Fhionna Moore, Shanice Taylor, Joanna Beaumont, Rachel Gibson, Charlotte Starkey

**Affiliations:** School of Social Sciences, Psychology, University of Dundee, Dundee, United Kingdom; University of Toronto, CANADA

## Abstract

**Objectives:**

To test for social structural effects on the gender paradox in suicidal behaviour.

**Methods:**

We analyzed newspaper reports of completed and attempted suicides in the Scottish city of Dundee during the mass movement of women into the paid labour force in the 19^th^ and early 20^th^ Centuries. We calculated rates of suicides per 100,000 of the male and female populations.

**Results:**

We found that the female suicide rate dropped during this time period, whereas there was only a significant reduction in attempted suicide amongst men.

**Conclusions:**

Our understanding, and action to prevent, suicide in men and women must take place in the context of our gendered social world.

## Introduction

More women than men are diagnosed with depression and women make more suicide attempts than men, yet more men than women die by suicide [1–7]. This gender paradox may stem, at least partly, from differences in the gendered social roles inhabited by men and women. These traditional roles of ‘homemaker’ and ‘breadwinner’ influence a variety of variables which may promote a gender paradox in suicide: experience of stress, likelihood of seeking help, and choice of suicide method.

In post-industrial westernised societies, men and women have traditionally been allocated to distinct social roles involving divergent responsibilities, expected behaviours and psychological traits, and stressors [8, 9]. Women, for example, have traditionally fulfilled the role of homemaker, taking responsibility for childcare, domestic work, and maintaining social relationships. Men have traditionally served as breadwinner, taking responsibility for financial provision.

Women report more regular stressful life events [10] and are more likely to suffer from stressors experienced by others [11] than are men. When women occupy the dual roles of employment and taking care of the home, they suffer a heavier stress burden than when managing a single role and are more likely to do so than are men [12]. Men are more likely to report financial stress [13] and report more frequent traumatic life events [14]. It is possible, then, that there are stressors unique to the male role of breadwinner which increase their risk of suicide [5].

Gendered social roles also determine the ways that men and women are expected to behave [2]. The traditional male stereotype is associated with aggression, proactivity, and violence, and the female stereotype with passivity, affection, and selflessness [5, 15]. The lethal and determined action required to commit suicide seems better situated in the proactive male role than the passive female role. This is also supported by the choice of more lethal and violent suicide methods by men than women [16]. Conversely, seeking help when experiencing distress is more aligned with the female than male role [17] and men are less likely than women to seek help when suffering from mental distress or illness [18], perhaps contributing to the higher male suicide rate. Evidence to date suggests that adherence to the masculine male gender role distinguishes between men who do and do not make a suicide attempt under stress such that men who more strongly identify with the masculine role are at greater risk, and that this is mediated by the effect of masculine role adherence on help seeking behaviour [19].

If gendered social roles contribute to sex differences in the suicide rate, we would expect the magnitude of the sex difference to change over time in accordance with temporal changes in gender equality in role occupation. At first glance, the data do not appear to support this. [20] reported that the movement of women into the paid labour force in the 1970s did not increase the female suicide rate, even amongst those women who juggled their employment with being a wife and mother. Conversely, there was a higher male suicide rate during the 1970s in communities with a higher rate of female labour force participation among married women with small children. One interpretation is that men were unable to cope with the loss of their role as the sole provider, resulting in a loss of self-esteem amongst men, particularly those who adhered most strongly to traditional gender roles [5]. Any contribution of gendered social-role occupation to suicide rate, then, is unlikely to be a simple additive effect, and will interact with prevailing norms, the extent to which a society has embraced gender equality, and local social ecology. Indeed, this is demonstrated by different effects of female labor force participation on the suicide rates of men and women in Canada between 1971 and 1981 [21]. In 1971, when prevailing societal views of married women in paid employment were largely negative, female labor force participation increased suicide risk for men and women. By 1981, however, with greater acceptance of women in work, female labor force participation decreased the risk of suicide for both sexes.

Here we test the hypothesis that the movement of working class women into the paid labour force will influence the sex difference in the suicide rate, at a time and location where working class women became the primary breadwinners. We extracted data from newspaper reports of completed and attempted suicides for the Scottish city of Dundee in the 19^th^ and early 20^th^ century. We chose this time and place as it provides a unique opportunity to explore a transition from an almost completely male workforce, to a majority female workforce in under 50 years. Women’s smaller size and lower wages favoured them as the primary employees in the city’s thriving jute mill industry [22], to the extent that Dundee became known as ‘She-Town’. We conducted exploratory analyses to determine whether the magnitude of sex differences in reports of suicide changed across the time period in which women moved into the city’s workforce.

## Method

### Sample

Reports of non-fatal suicide attempts and completed suicides (‘events’) for Dundee were extracted from articles published in local newspapers between January 1^st^ 1844 and December 31^st^ 1950. This provided a sample of 702 events (female n = 274, male n = 428). Our methodology received full approval from the University of Dundee Research Ethics Committee.

### Time period

There were no records available for Dundee newspapers published prior to 1844, so this marked the earliest point of our search. This also coincided with the movement of women into the workforce as women are first reported to be working in the city’s mills in the 1840s, with numbers increasing until the early 20^th^ Century when 70% of mill workers were women [22]. By the mid 20^th^ century, the jute industry was in decline. Therefore, we analysed data extracted from archives covering 1844–1950.

### Data extraction

Editions of local Dundee newspapers were searched via the online British Newspapers Archive (http://www.britishnewspaperarchive.co.uk). All articles that were published in all newspapers published locally during the time-period were searched for the key words “suicide” and “Dundee”. Records were returned for 10 publications: Dundee Advertiser (records returned between 1861–1899), Dundee Courier (records returned between 1844–1955), Dundee Evening Post (records returned between 1900–1905), Dundee Evening Telegraph (records returned between 1877–1950), Dundee People’s Journal (records returned between 1858–1930), Dundee Weekly News (records returned between 1879–1892), The Dundee Yearbook (records returned in 1901), Dundee, Perth and Cupar Advertiser (records returned between 1839–1864), Northern Warder (records returned between 1841–1869), and Broughty Ferry Guide and Advertiser (records returned between 1889–1892).

All articles returned by the search were screened to ensure that they reported a relevant event and, if so, that the event occurred within the city of Dundee. Some records, for example, were returned for events that occurred in neighbouring areas and these were excluded from the dataset. This was because we were interested specifically in the shifting gender equality of paid employment and fulfilment of the breadwinner role under the unique circumstances of Dundee city’s jute mills. Additionally, some articles gave only a street name and in these cases, the street name was researched to ensure that it was a Dundonian street at the time using historical ordnance survey maps, accessed via Dundee Central Library. If it was not possible to identify the street in Dundee, the event was excluded from the dataset.

We included as events those deaths that were labelled as “suicides” and “suspected suicides”. All entries were reviewed to ensure that the event was not replicated in other publications. Where there were no events reported for a year, we coded the year as having 0 reported events for both male and female suicides. This was the case for 1860, 1871, 1916–17, and 1920–21. As there were also no data for the years 1923–1932 and 1934–1942, we excluded the small number of records retrieved after 1922 (n = 2).

For each event we recorded the gender and event outcome (non-fatal or fatal).

### Statistical analyses

To compare sex differences in attempted and completed suicides over time, we first calculated the number of suicides per 100, 000 of the male and female populations. This controlled for the female biased sex ratio in Dundee at the time. Male and female population sizes were obtained from census records reported by [22]. As a census was taken every 10, 20 or 30 years, total rates of male and female events, and of male and female attempted and completed suicides, were calculated for the following periods: 1844–1850 using population sizes from the 1841 census, 1851–1860 using population sizes from the 1851 census, 1861–1880 using population sizes from the 1861 census, 1881–1910 using population sizes from the 1881 census, 1911–1920 using population sizes from the 1911 census, and 1921–1922 using population sizes from the 1921 census. The sex ratio for rates of total events, and attempted and completed suicides, were calculated (male rate/female rate). When rates were zero for both males and females in any given year, the ratio was treated as 1.

All rates and ratios were positively skewed so were log transformed.

We tested whether rates of each type of event per 100, 000, and sex ratios of each type of event, changed over time first by entering each as the dependent variable in a time-series model using the expert-modeller function of SPSS. This function estimates Autoregressive Integrated Moving Average (ARIMA) models for time series data in order to calculate the following parameters that result in the model of best fit: autoregressive order (‘p’: the number of lagged units on which the dependent variable is regressed), moving average order (‘q’: the number of units averaged together to ‘smooth’ the time series data), and degree of differencing (‘d’: the number of times a data point has had past data points subtracted from it).

To further explore any trends over time, we entered each rate and ratio as dependent variables in a multivariate analysis of variance with ‘time period’ (early, mid, and late, based on tertiles) as the independent variable.

## Results

In total there were 147 attempted suicides by women, 127 completed suicides by women, 178 attempted suicides by men, and 250 completed suicides by men.

### Sex differences over time in numbers of completed and attempted suicides

In time-series analysis, ARIMA (0, 0, 0) was identified as the best fit model for the rate of completed male suicides and sex ratios of total events, attempted suicides, and completed suicides. This indicates that there were no significant temporal patterns for these models. ‘Simple’ models were identified as best fit for the rates of total female and male events, and of attempted male and female suicides (i.e. there were zero orders of autoregression, one order of differencing, one order of moving average, and no constant). A ‘Holt’ model was identified as best fit for the rate of completed female suicides (i.e. there were zero orders of autoregression, two orders of differencing, and two orders of moving average).

**Fig 1:**
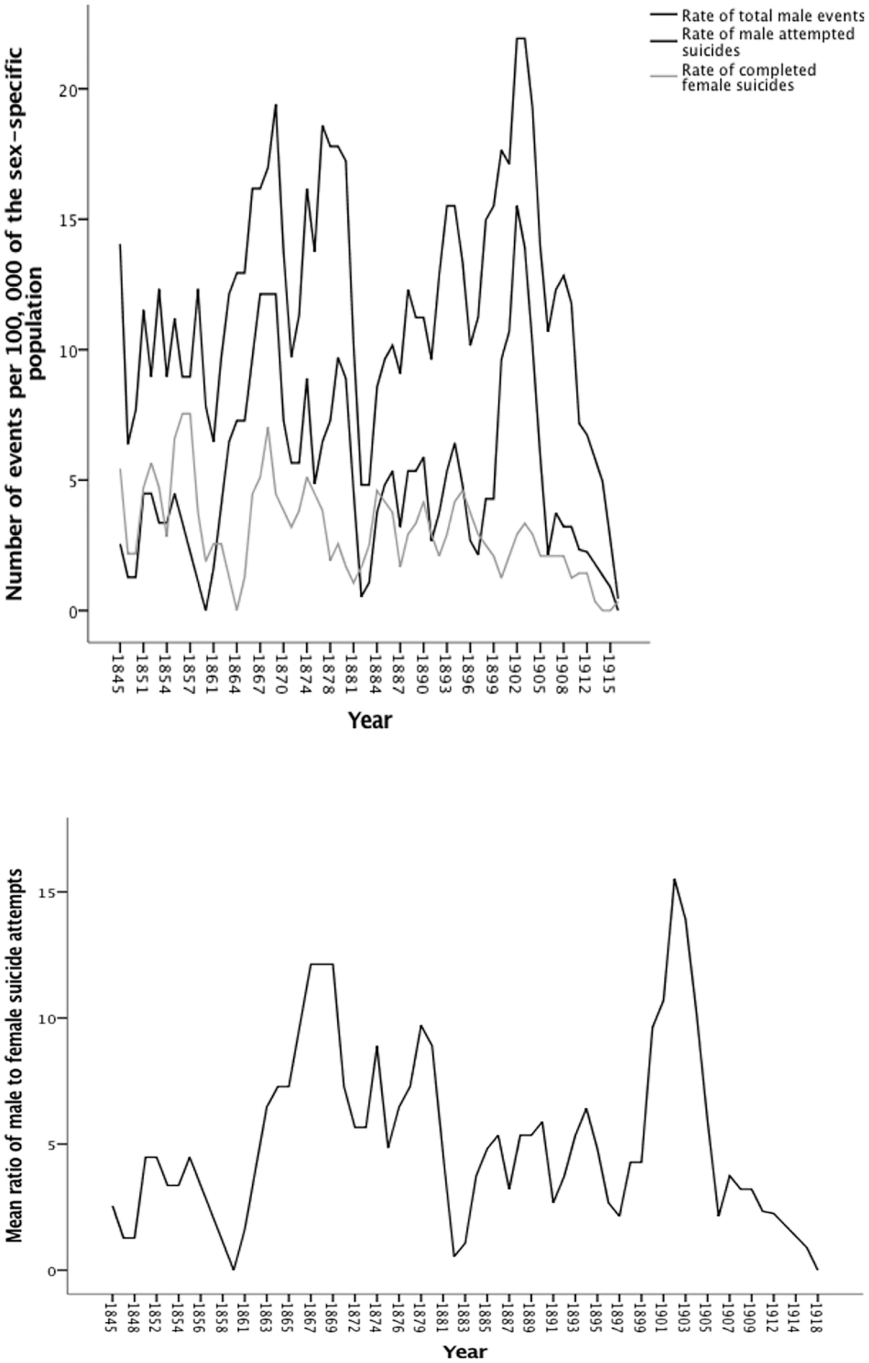
Significant trends over time in (a) rates of total male events, male suicide attempts and completed female suicides and (b) in the ratio of male to female attempted suicides. Rates are based on 3-year moving averages.

There were significant trends over time in rates of total male events (stationary R^2^ = 0.3, Ljung-Box Q(17) = 31.66, p = 0.017), male suicide attempts (stationary R^2^ = 0.36, Ljung-Box Q(17) = 31.28, p = 0.018), and completed female suicides (stationary R^2^ = 0.83, Ljung-Box Q(17) = 31.3, p = 0.012). There was also a significant trend over time in the ratio of male to female attempted suicides (stationary R^2^ < 0.01, Ljung-Box Q(18) = 38.76, p = 0.003). All significant trends were negative (i.e. rates decreased over time). Trends in all other rates and sex ratios were non-significant (p > 0.06). Fig 1a shows the trends over time in the significant rates of events, and Fig 1b shows the significant trend over time in the ratio of male to female attempted suicides.

**Fig 2:**
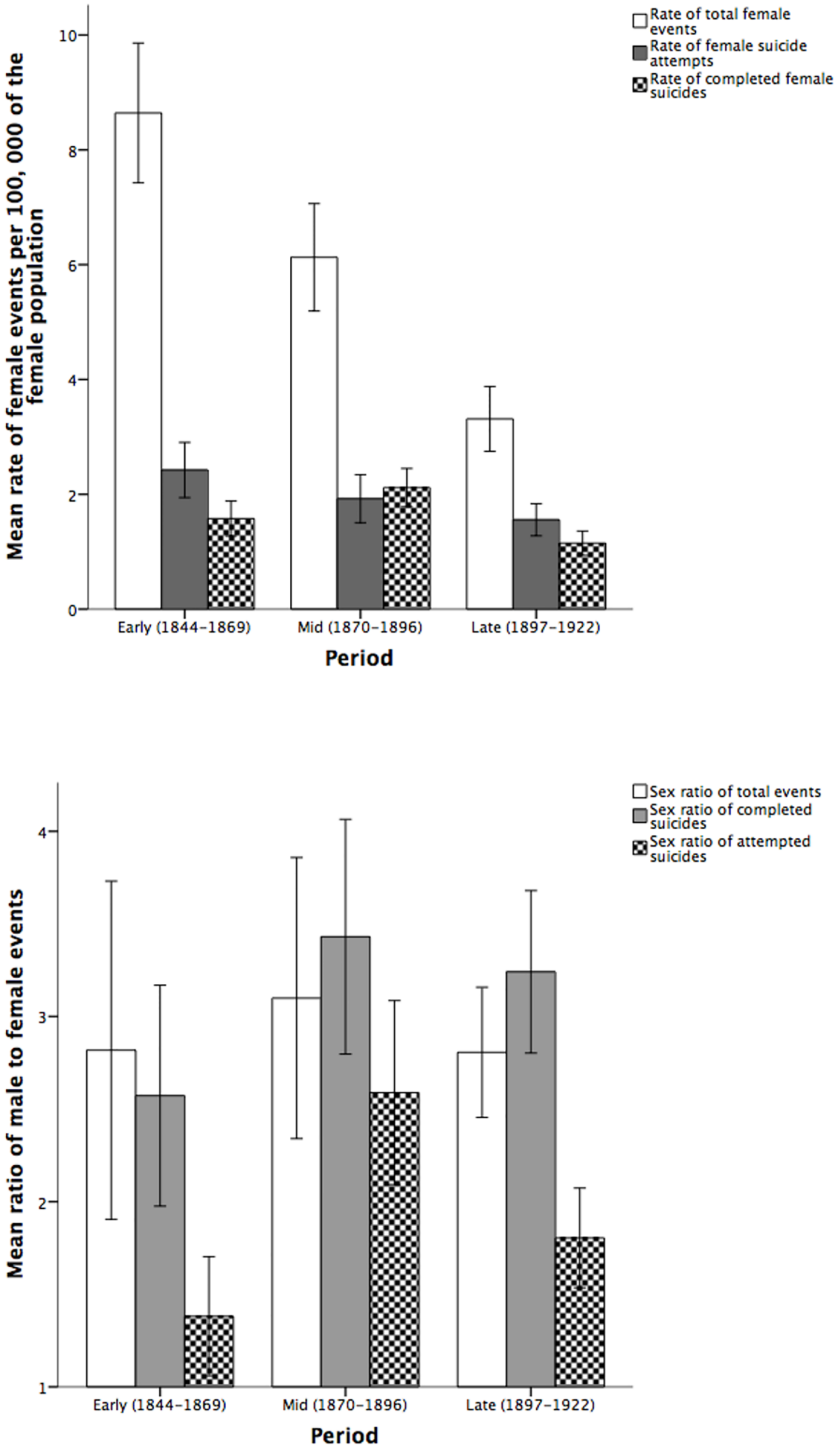
(a) Rates (per 100, 000 of the female population) of total female events and female completed and attempted suicides, total events, and (b) male to female ratios of total events, and attempted and completed suicides.

To further explore these changes over time, we fit multivariate analysis of variance models with ‘period’ as the fixed effect. There were significant effects on the rates of total female events (F(2, 34) = 11.54, p < 0.001, partial eta-sq = 0.4), female suicide attempts (F(2, 34) = 10.44, p < 0.001, partial eta-sq = 0.38), and completed female suicides (F(2, 34) = 8.5, p = 0.001, partial eta-sq = 0.33). There were also significant effects on all 3 sex ratios: total events (F(2, 34) = 9.39, p = 0.001, partial eta-sq = 0.356) suicide attempts (F(2, 34) = 7.63, p = 0.002, partial eta-sq = 0.31), and completed suicides (F(2, 34) = 8.44, p = 0.001, partial eta-sq = 0.33). Post-hoc tests revealed that there were significant differences in total female events between the early and mid (p = 0.006) and early and late (p < 0.001) time periods; significant differences in female suicide attempts between the early and mid (p = 0.001) and early and late (p < 0.001) time periods; significant differences in female completed suicides between the early and late (p < 0.001) and mid and late (p = 0.031) time periods; significant differences in the sex ratio of total events between the early and mid (p = 0.003), and early and late (p < 0.001) time periods; significant differences in the sex ratio of attempted suicides between the early and mid (p = 0.001) and early and late time periods (p = 0.004) time periods; significant differences in the sex ratio of completed suicides between the early and mid (p = 0.021) and early and late (p < 0.001) time periods. All other differences over time were non-significant (p > 0.6). Fig 2a shows the significant differences over time in rates of total female events, female suicide attempts, and completed female suicides. Fig 2b shows the significant differences over time in the sex ratios of total events and completed and attempted suicides.

## Discussion

Here we show that the frequency of newspaper reports of male and female suicidal behaviour changed during industrialisation of the Scottish city of Dundee when working class women became the primary breadwinners. We framed our analysis in the context of the gendered socialisation of men and women which may influence the experience of stressors, psychological factors related to suicide, and the likelihood of seeking help when experiencing distress. We argued that these may, in turn, contribute to the sex difference in suicide. We also noted, however, that suicide rates of men and women may not simply merge as societal gender equality increases as any potential relationships are situated in a complex web of societal and ecological forces. For this reason, our work was largely exploratory and capitalised on a time period when women became the primary breadwinners. Indeed, rather than a straightforward switch in the gender suicide paradox as roles shifted, we found evidence for the rates of female attempted and completed suicide and male attempted suicide to reduce significantly as female participation in the workforce and occupation of the breadwinner role increased. The ratio of male to female suicide attempts and completed suicides showed a curvilinear trend over time with relatively more men than women attempting and completing suicide across the time period, but with a more marked difference in the middle time period (1870–1896).

We interpret our results in the context of an interaction between traditional and changing roles for men and women, as well as the physical environment. Working class Dundee at the time suffered from profound deprivation. There were extremely high levels of overcrowding and infant mortality, air quality was poor, and malnutrition was high [22, 23]. It is against this backdrop, then, and the history of traditional male and female roles and socially enforced expectations, that we must interpret the reduction in reports of suicide attempts across the study time period.

As has been suggested by [5] and [20], loss of status as the primary provider in the household may contribute to male suicide when women move into the workforce. This may have been particularly salient in Dundee during industrialisation, as working class women not only became primary breadwinners, but were also reported to take on male traits such as drunkenness and brawling, and while Dundee became known as ‘She Town’, Dundee’s men became known as ‘kettle boilers’ for their role in the home [22]. This was likely a time of profound adjustment for men, with important potential consequences for their mental wellbeing and risk of suicide. It is interesting, then, that the rates of male suicide attempts decreased over the time period. Perhaps there were stressors associated with the masculine breadwinner role which increase suicide risk in men, and removal from this role reduced exposure to this risk. Interestingly, the number of men relative to women who attempted or completed suicide was roughly equivalent at the beginning and end of the time period, with a significant peak at the mid-point. This is consistent, at least partly, with a model in which the changing role of men results in increased risk of mental distress. That is, it is tempting to speculate that this was a response to the movement of women into the labor market in the latter half of the nineteenth century resulting in an increase in male suicide, which then returned to normal levels as the social change became embedded in the early twentieth century. It is not clear, however, why this effect is only apparent when comparing the sex ratio of suicide rates, and not when looking at the male suicide rate in its own right.

We did not find support that breadwinner-specific stressors increased the suicide rate in women when they moved into the role. Rate of reports of attempted and completed female suicides reduced over time, suggesting a reduction in stressors despite the dual roles of mother and breadwinner fulfilled by many women [22]. It seems that, at least according to newspaper reports at the time, adoption of the breadwinner role reduced the risk of suicide in women. Perhaps becoming the financial provider in the household increased power and status in the home and wider community, which had positive benefits on mental wellbeing that outweighed the stressors of fulfilling two roles. As we discussed in the Introduction, the impact of female labor force participation on suicide depends upon social norms around women’s paid employment [20, 21]. The predominantly female workforce and associated rapid change in social norms around women in employment [22, 23] and the heavily biased female sex ratio, coupled with the local socioecology may have favoured a reduction in suicide rate of men without a concurrent increase in the suicide rate of women. This may not be the case for other populations under different circumstances.

It is important to note that we did not measure suicide rate here, but only rate of reports of suicidal behaviour. The frequency and nature of such reports were likely influenced by the interests and fashions of the time. We have assumed that reports reflect a reasonably accurate proxy of actual rate, but it is possible that they are a better proxy of, for example, public interest. This, in itself, is an interesting topic for investigation but was not the subject of our study. It is also not clear to what extent the events reported in local newspapers are a reflection of patterns amongst the working class specifically, who were the population of interest in terms of shifting gender roles, or of the full spectrum of social status more broadly. It is also possible that many suicides went unreported if they were treated as, for example, accidents. We suggest that future studies could conduct analyses similar to those reported here but using alternative sources of data on suicide rates, should test changes in the sex difference in suicide rate in different populations and ecologies, and should attempt to identify psychological mediators of gender roles and suicide rates (e.g. the use of violent suicide methods by men and women in breadwinner and homemaker roles).

In conclusion, our results demonstrate that the male and female suicidal behaviour, as measured by reports in local newspapers, shifted with the movement of working class women into the workforce. There was evidence for reduction in attempted suicides by both men and women and completed suicides in women. The gender suicide paradox increased in the mid-point of our study time period, and returned to earlier levels by the 1920s. Therefore, contrary to a simple shift in suicide rates with a corresponding shift in gender roles, we argue that gender differences in suicide rates are the product of complex interactions between traditional and prevailing norms and expectations around gender, and the demands and affordances of new and contemporary role occupation.

## Supporting information

S1 Dataset This dataset contains all data analysed and reported in the study. (XLSX)
